# Benefiting from the past: establishing in vitro culture of European beech (*Fagus sylvatica* L.) from provenance trial trees and seedlings

**DOI:** 10.1186/s13007-025-01350-3

**Published:** 2025-03-07

**Authors:** Virginia Zahn, Alexander Fendel, Alice-Jeannine Sievers, Matthias Fladung, Tobias Bruegmann

**Affiliations:** Thuenen Institute of Forest Genetics, Sieker Landstrasse 2, 22927 Grosshansdorf, Germany

**Keywords:** Antibiotics, Biotechnology, Contamination control, Forest trees, Phytohormones, Recalcitrance, Woody plants

## Abstract

**Background:**

European beech (*Fagus sylvatica* L.) is distributed across diverse climate conditions throughout Europe. Local adaptations, such as drought tolerance, could become crucial for maintaining beech populations facing climate change. In vitro culture offers a promising tool for preserving and propagating valuable genotypes and provides a basis for biotechnological research, although establishing and propagating recalcitrant beech in vitro is difficult. To the best of our knowledge, this study is the first to use beeches from a provenance trial to establish in vitro cultures, aiming to capture a wide genetic spectrum and investigate provenance-specific suitability for in vitro cultivation. In addition, a high-throughput method using seedlings has been developed to increase the success of establishing in vitro cultures of a provenance.

**Results:**

Actively growing shoots from 22 field-grown provenances were obtained for in vitro establishment. After 12 weeks, shoot formation on shoot tips and nodal segments was induced in 13 provenances (57%), with success rates ranging from 3 to 80%, significantly influenced by the provenance and sampling date of the branches. Combining one harvest each in February and May resulted in the highest shoot formation rate (18%). However, after two years, stable micropropagation was achieved for a single genotype. In the second approach, whole shoots or shoot tips from seedlings were used for in vitro establishment, achieving shoot formation rates between 38 and 94%. Bacterial contamination during establishment was controlled through antibiotic application. Using culture medium without phytohormones improved initial leaf flush on shoot tips within the first 8 weeks of in vitro culture. Phytohormone-supplemented media were needed for shoot multiplication and prolonged in vitro culture. Cultures of 25 genotypes were maintained for up to two years. The viability of in vitro shoots was maintained by supplementing the medium with FeNaEDTA, MgSO_4_, and glucose. Some genotypes showed enhanced performance on sugar-free media with increased light intensity, which reduced bacterial outgrowth.

**Conclusion:**

With the technical approaches presented here, we provide starting points for the establishment of beech cultures from various types of starting material, as well as for further method improvement for establishment and long-term cultivation.

**Supplementary Information:**

The online version contains supplementary material available at 10.1186/s13007-025-01350-3.

## Background

European beech (*Fagus sylvatica* L.), a member of the beech family (*Fagaceae*) and hereafter named beech, is one of the most important deciduous tree species of the natural vegetation of Central European forests. Despite their silvicultural and ecological value for native wildlife, beech trees are economically important for forestry [1–3]. Beech is naturally distributed over large parts of Europe. Beech populations, which have adapted to a wide range of habitats and climatic conditions, extending from southern Sweden to Sicily and from northwestern Spain to Ukraine [4]. The large ecological amplitude is reflected in local adaptations, e.g., in terms of leaf shape or response to drought and high or low temperatures [5, 6]. Those local adaptations have been studied within provenance trials since the 1950s but have become increasingly relevant in light of climate change [7–9].

Beech prefers a temperate climate with moist summer conditions and moderately moist soil [10], making it vulnerable to climate change [11–13]. Central European stands are struggling with a long-term decline in growth rate and an increase in crown defoliation, disease symptoms, and mortality (beech dieback) triggered by the summer droughts of 2018–2019 [14–16]. Beech stands in northern Spain are threatened by major losses or even regional extinction [17].

Breeding resilient beech trees is challenged by the species' long generation cycle, which can take up to 50 years before fructification occurs [4]. Although the postglacial spread of beech has been anthropogenically influenced, this species has not been domesticated as a crop plant [18]. As a result, populations continue to exhibit significant genetic variability and phenotypic plasticity, which can be advantageous for adapting to environmental challenges such as drought stress [10, 19–22]. The existing genetic diversity, especially of those populations that have already adapted to rather warm and dry climates, could become crucial for the survival of beech under climate change [23, 24].

In vitro culture provides an opportunity to preserve and propagate valuable and possibly endangered plant genotypes. Given the poor rooting ability of beech stem cuttings [25], in vitro culture can serve as an alternative method for vegetative propagation. However, beech trees are known to be recalcitrant to in vitro micropropagation since the early 1980s [26]. Thus, publications on the *in vitro* culture of *F. sylvatica* or the related species *Fagus grandifolia* and *Fagus orientalis* are rare and were published mostly in the 1980s and 1990s [27–31]. Nevertheless, the knowledge attained in the past has the potential to guide the development of new approaches that are urgently needed due to new ecological challenges and interest due to new technological advances in various areas, e.g., genomics and biotechnology [32–35].

For the success of in vitro culture, the nutrient composition of the basal medium is one of the crucial factors [29, 30]. For trees such as beech, the woody plant medium (WPM) with relatively low nutrient concentrations is commonly used [28, 29, 36–38]. However, Gresshoff-Doy (GD) medium has demonstrated improved survival rates for in vitro shoots derived from a 10-year-old beech genotype [29]. Sucrose is widely used as carbohydrate source [31, 37, 39, 40], but glucose has demonstrated genotype-specific advantages for both shoot multiplication and length [40].

Micropropagation of trees can be achieved by either organogenesis or somatic embryogenesis. Compared with organogenesis, somatic embryogenesis in *F. sylvatica* is more difficult to trigger and less efficient in terms of plant regeneration [38, 41, 42]. During organogenesis, new shoots develop from existing or de novo formed meristems [43]. This process is strongly regulated by the addition of phytohormones, typically a combination of cytokinins and auxins, to the cultivation medium [44]. For beech in vitro cultivation, the cytokinin 6-benzylaminopurine (BAP) is commonly used at concentrations ranging from 0.44 to 5 µM, often in combination with lower concentrations of auxins [28, 30, 45, 46]. The optimal BAP concentration depends on the type of explant and developmental stage. Closed buds typically require higher concentrations initially (4.5–5 µM BAP) while the following shoot multiplication occurs with 0.9 µM BAP [29, 46]. For young beech seedlings, experiments were carried out with 0.89–4.44 µM BAP to induce shoot formation [45]. For the closely related species *F. grandifolia*, which uses both open buds and shoot tips from root sprouts or seedlings for in vitro establishment, 0.89 µM BAP resulted in the most robust shoot growth. In saplings aged three months to two years, shoot elongation of up to 1.8 cm and a shoot multiplication rate of 1.3 within 12 weeks of in vitro culture were reached [30]. To our knowledge, a protocol for establishing in vitro cultures from shoot tips of European beech seedlings has not yet been published. To obtain a comprehensive picture of the influence of phytohormones, Vieitez et al. reported that the combination of 2.2 µM BAP, 2.89 µM NAA, and 9.1 µM Zeatin was effective for the micropropagation of beech shoots derived from embryonic axes [28].

Also the explant source plays a crucial role during culture establishment. The age of the plant affects in vitro shoot formation, regeneration rates, and ease of subculturing [27, 29, 30, 36]. In particular, mature genotypes often prove difficult to cultivate in vitro, prompting experiments with rejuvenated material obtained through grafting or root sprouts, which resulted in an increase in viable genotypes from juvenile and rejuvenated sources compared to older trees [29, 30].

Prolonged growth of buds in situ on the tree before in vitro establishment, i.e., harvesting the material in early spring instead of late autumn or winter, results in greater shoot formation. In weighing the trade-off between extended growth in situ and the associated increased risk of contamination, Meier and Reuther recommended harvesting closed buds for in vitro establishment in February [29]. In contrast, Barker et al. reported that open buds from mature *F. grandifolia* trees sprouted under controlled conditions after harvesting in winter could be successfully used for establishing in vitro cultures [30]. When seeds are used as explants, the loss of explants to contamination can be controlled by using embryonic axes instead of whole embryos, resulting in adventitious bud formation rates of up to 35% [28, 38].

In addition to in vitro establishment and micropropagation and as the final piece of the puzzle, root formation is necessary to stabilize a culture and enable its transfer to *ex vitro* conditions, aiming to facilitate its use for experiments or forestation. Rooting of beech microcuttings can be initiated by an indole-3-butyric acid (IBA) dip (0.5–4.92 mM for 30 s to 2 min), producing rooting rates of up to 90% [28, 29, 36, 37].

The knowledge attained in the past underscores the complexity of in vitro culture establishment in *Fagus* species because each variable—basal medium, carbon source, phytohormones, explant age, and explant type—must be carefully optimized to achieve sustainable growth. Even if all these variables can be optimized, the plant's genotype appears to be a key factor with the greatest impact on in vitro performance [28–30, 47]. Genotype- or provenance-specific differences in performance have been shown, e.g., for beech with respect to adventitious bud regeneration, for Kashmir trees (*Gmelina arborea*) with respect to shoot formation, or for olive trees (*Olea europaea*) with respect to suitable medium composition [28, 48, 49]. Starting with a diverse gene pool during establishment can therefore be beneficial in finding a well-growing in vitro line.

Local variations of beech across its distribution range have been investigated in provenance trials for many years [7–9] but these have not been utilized for in vitro establishment. In this study, we capitalize on the high genetic diversity of beech populations to explore their potential for in vitro cultivation. We considered different genotypes aged between three and 24 years, derived from 23 provenances across the distribution area, and grown in a provenance trial for the establishment of an in vitro culture (Experiment I). Shoot tips and nodal segments of actively growing shoots were tested for their in vitro cultivability from branches collected in February with closed buds to minimize the contamination rate. Due to the unexpectedly low bud break rate of February samples, branches with slightly open buds were harvested in May [29].

In a second approach, three-week-old beech seedlings derived from a German provenance were used as a young material source to increase the growth response under in vitro conditions and reduce the contamination risk (Experiment II). On the basis of positive experiences, Experiment III was conducted using young seedlings from a second German provenance. To control bacterial contamination, this study tested a modified sterilization protocol and introduced antibiotic supplements. To our knowledge, the use of antibiotics in beech in vitro culture has not yet been described. These measures, alongside adjustments in media composition, aimed to prevent the rapid decline in explant vitality and suggest a more sustainable approach for long-term culture maintenance, addressing the recalcitrance of beech cultures.

These approaches contribute to assessing provenance-dependent variations in beech responses to in vitro culture, improving the success rates of in vitro establishment and clonal propagation. This could enable future forestry applications of such propagation systems, as well as the further transfer of novel biotechnological methods to beech and other tree species.

## Materials and methods

### Plant material

Two main types of beech explants were used for shoot formation *in vitro*: plant material from provenance trial trees aged 3–24 years (Experiment I, designated E I) and three-week-old seedlings (Experiment II [E II] and Experiment III [E III]). Details are described for each experiment.

### Plant growth chamber (PGC) conditions

Bud break on branches of provenance trial trees (E I), seed germination (E II and E III), and in vitro culture establishment (E I–E III) occurred under the same PGC conditions to minimize stress during establishment. These standard conditions included a 16-h photoperiod, 22/18 °C day‒night cycle, and 70–80 µmol m^−2^ s^−1^ light intensity in a PGC (Weiss Technik, Reiskirchen, Germany).

### Culture media and micropropagation

All media used during in vitro culture establishment (E I–E III) were based on McCown Woody Plant Medium including vitamins (WPM) [50] with 2% sucrose and 0.65% agar, with variable concentrations of phytohormones (Table 1). The phytohormone concentrations in medium M1 were derived from Chalupa [45]. The concentrations in medium M2, as well as the concept of the stepwise increase in BAP concentrations in M2.1 and M2.2, were adopted from Barker et al. [30]. Medium M3 is based on Cuenca et al. [37]. All the medium ingredients were supplied by Duchefa Biochemicals (Haarlem, The Netherlands). The pH was adjusted to 5.6 with 1 M NaOH before autoclaving.

**Table 1:** Phytohormone concentrations of the culture media used for beech in vitro culture

Medium	Phytohormone concentration [µM]			
	BAP	NAA	IAA	Zeatin
M0	0	0	0	0
M1	2.2	0	0	0
M2	0.89	0.27	0	0
M2.1	2.2	0.27	0	0
M2.2	3.55	0.27	0	0
M3	2.2	0	2.9	9.1

BAP, 6-Benzylaminopurine; NAA, 1-Naphthaleneacetic acid; IAA, Indole-3-acetic acid

In E III, medium M0 was supplemented with 500 mg/L cefotaxime, 50 mg/L gentamicin, and 50 mg/L streptomycin (designated M0-AB). To improve the prolonged culture of microcuttings, medium M2 was supplemented with 50 µM FeNaEDTA and 400 µM MgSO₄ (designated M2 +), after chlorosis and arrested shoot elongation (indicated by resting bud formation) were observed in microcuttings from E I and E II. According to a personal note from J.-P. Schnitzler’s group at the Helmholtz Center Munich, the increase in Fe and Mg reduces chlorosis and inhibits resting bud formation, symptoms often associated with deficiencies in magnesium or iron [51].

The microcuttings were transferred to fresh medium every 6 weeks. When in vitro shoots of a genotype showed chlorosis, resting bud formation or excessive bacterial growth on M2 +, , the medium was switched to M2 +  woS according to our experiences from other research projects. The medium M2 +  woS corresponds to M2 +  but does not contain sucrose.

As we observed limited elongation and multiplication on M2 + and M2 + woS, glucose was tested as a carbon source, as described in Cuenca and Vieitez [40]. In vitro shoots from each genotype were cultured on M2 + medium supplemented with 2% glucose (M2 + G), and growth was visually assessed.

A reduction in chlorosis and growth arrest of in vitro cultures was observed in microcuttings growing on sugar-free M2 + woS. Improved growth on sugar-free media typically only works at light intensities that would cause photo stress on sugar-containing media during mixotrophic growth [52]. Therefore shoots that have been cultivated on medium M2 + G in prior were transferred to a reduced light intensity of 30 µmol m^−2^ s^−1^.

### Experiment I

#### Plant material

Differences in the ability of beech provenances to form shoots in vitro were investigated via provenance trial trees. The Berlin Climate Forest is a provenance trial with beech trees from different European provenances. The site is located in the sandy Berlin forest “Grunewald” (52°29′12.0"N, 13°15′05.4"E) and thus in the temperate transition zone between maritime and continental climates. The provenance trial was established in 2013 by M. Forstreuter (Freie Universität Berlin, Berlin, Germany). The trees were previously grown from seeds in pots starting in 1998 and then gradually planted as young trees at the site. Its experimental layout is a randomized block design with 25 trees per block and up to three replicates. The plantings were made in rows with 2 m × 1 m spacing.

**Fig. 1:**
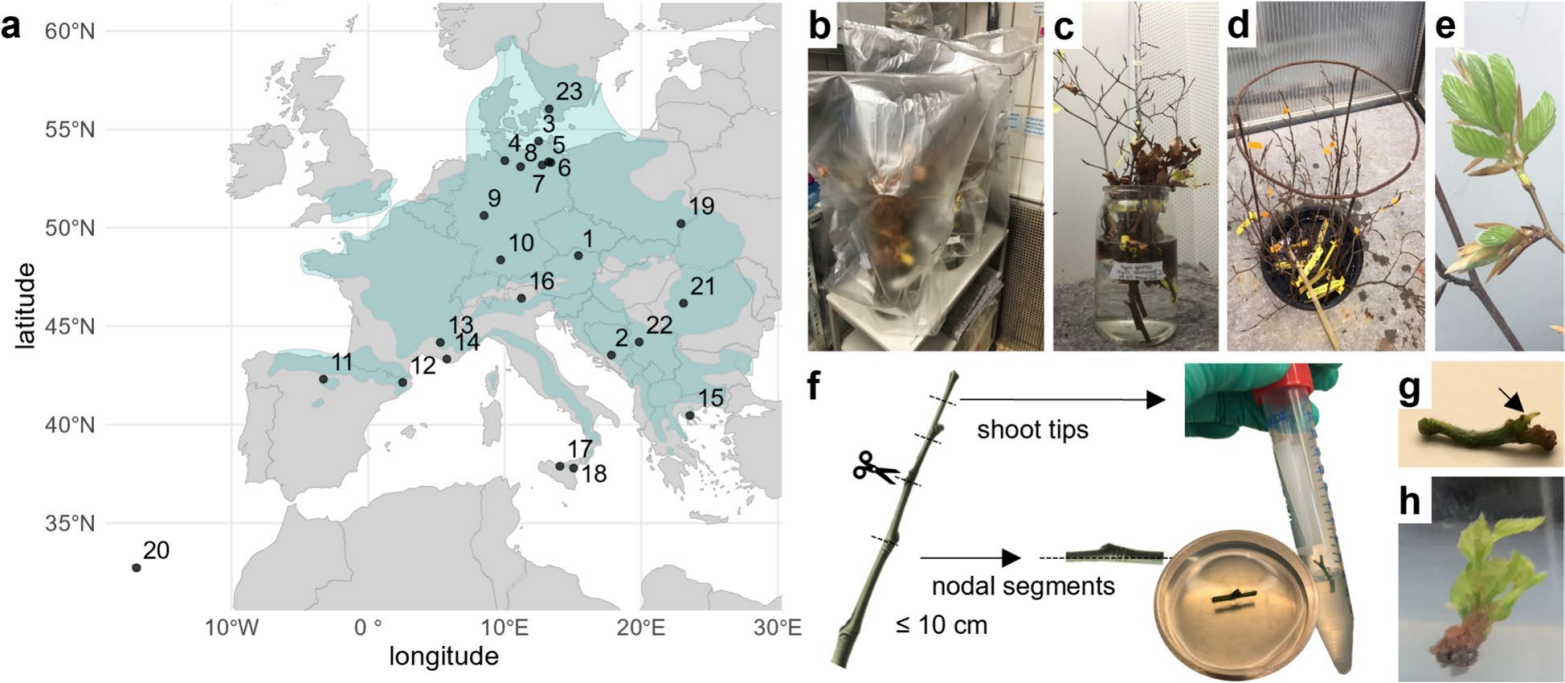
In vitro culture establishment using branches from the provenance trial ‘Berlin climate forest’. **a** Provenances (black dots) are labeled by number according to Table 2. The distribution area of *F. sylvatica* is highlighted in blue. **b** The branches were stored at 4 °C until bud break was initiated under **c** plant growth chamber conditions in water-filled glass vessels or **d** greenhouse conditions with constant water flow until **e** actively growing shoots emerged from both apical and axillary buds. **f** Shoot tips and nodal segments were taken from actively growing shoots of up to 10 cm in length. After surface sterilization, the nodules were cut longitudinally and placed on medium M1 with the cut side down. The shoot tips were placed upright in the same medium. Four weeks after in vitro establishment, **g** nodal segments with buds and shoot tips were transferred to M2. The illustration is not to scale. **h** Shoot formation after 4 weeks of cultivation on M1 and 8 weeks on M2

Branch samples from 3- to 24-year-old beech trees were collected at two different occasions to assess the effects of bud status on contamination and bud break ability (Additional file 1). In February 2022, branches with closed dormant buds were harvested to minimize contamination. After branches sampled in February initially presented a low capacity for bud break, a second sampling was performed in May 2022 with slightly opened buds on branches. If a provenance was sampled at both time points, different trees were selected for each sampling. Overall, branches of 65 trees from 23 provenances across 11 countries were collected (Table 2; Fig. 1a).

**Table 2:** Beech provenances and explants obtained for in vitro culture establishment (Experiment I)

No	Country	Provenance	Age [years]	N sampled trees	Sampling month	Bud break conditions	N explants	Σ N explants	N contamination	N shoots
1	AT	Dobra	20	1	Feb	PGC	0	27	6	2
				2	Feb	GH	27			
2	BA	Bugojno Vranica-Bistrica	17	1	Feb	PGC	0	21	17	0
				1	May	GH	21			
3	DE	NP Mecklenburg-Western Pomerania	15	1	Feb	PGC	0	9	8	1
				1	Feb	GH	0			
				2	May	GH	9			
4	DE	Busschewald	24	1	May	GH	35	35	25	1
5	DE	NP Mueritz Serrahn	15	1	May	GH	12	12	2	8
6	DE	Heilige Hallen	19	1	Feb	PGC	0	64	32	2
				1	Feb	GH	30			
				2	May	GH	34			
7	DE	Flecken-Zechlin	21	1	Feb	PGC	0	40	29	4
				1	Feb	GH	21			
				2	May	GH	19			
8	DE	Luechow	24	1	May	GH	40	40	36	0
9	DE	Westerwald	3	1	Feb	PGC	7	7	3	0
				1	Feb	GH	0			
10	DE	Swabian Alb	17	1	May	GH	33	33	2	1
11	ES	Rábanos, Burgos	13	1	Feb	PGC	0	21	21	0
				1	Feb	GH	0			
				2	May	GH	21			
12	ES	NP Volcanic Zone La Garrotxa	17	1	Feb	PGC	0	45	13	4
				1	Feb	GH	22			
				2	May	GH	23			
13	FR	Mont Ventoux	22	1	Feb	PGC	10	52	14	0
				1	Feb	GH	42			
14	FR	St Baume	22	1	Feb	PGC	0	37	35	0
				1	Feb	GH	0			
				3	May	GH	37			
15	GR	Chalkidiki	13	1	Feb	PGC	12	24	14	0
				2	Feb	GH	12			
16	IT	Mendelpass 1300 m	5	1	Feb	PGC	0	4	1	1
				1	Feb	GH	0			
				2	May	GH	4			
17	IT	Madonie	3	1	Feb	PGC	0	32	26	0
				1	Feb	GH	0			
				1	May	GH	32			
18	IT	Etna	18	1	Feb	PGC	0	72	29	14
				1	Feb	GH	35			
				1	May	GH	37			
19	PL	Oleszyce	14	1	Feb	PGC	0	50	26	1
				1	Feb	GH	28			
				1	May	GH	22			
20	PT	Madeira	8	1	Feb	PGC	0	27	0	3
				1	Feb	GH	27			
21	RO	Alka-Iulia	17	1	Feb	PGC	0	19	15	1
				1	Feb	GH	0			
				1	May	GH	19			
22	RS	Cer	17	1	Feb	PGC	0	0	0	0
				1	Feb	GH	0			
23	SE	Skaeralid	18	1	Feb	PGC	0	20	6	2
				1	Feb	GH	0			
				2	May	GH	20			
Total				65			691	691	360	44

Branches were sampled in February (Feb) or May in the Berlin Climate Forest. Bud break on branches took place under plant growth chamber (PGC) or greenhouse (GH) conditions. Nodal segments and shoot tip samples of actively growing shoots were introduced into in vitro conditions. The age and number of trees sampled per provenance are indicated. Zero explants indicated that bud break on the sampled branches had failed. The initial number of explants, the number lost to contamination, and the number of shoots newly developed after 12 weeks under in vitro culture conditions are given. Country abbreviations according to ISO 3166 1 alpha2

NP, National park

Actively growing shoots were used for in vitro establishment according to Barker et al. [30]. After harvest, branches sampled in February were stored in water at 4 °C in the dark and covered with plastic bags to prevent dehydration for one to two months (Fig. 1b). To initiate bud break, the branches were placed in water-filled glass vessels under standard PGC conditions, and water was replaced three times a week (Fig. 1c). Alternatively, branches were placed under greenhouse (GH) conditions without temperature control but with constant water exchange (Fig. 1d). Branches sampled in May were directly transferred to GH conditions. The GH had consistently higher temperatures than the PGCs because of weather conditions. Cut ends of branches in both the PCG and greenhouse were re-cut three times a week.

Shoots were allowed to grow to a length of 10 cm after bud break to obtain a greater number of explants for each provenance (Fig. 1e). Actively growing shoots developing from both apical or axillary buds were cut into shoot tips and nodal segments approximately 1 cm in length after removing the leaves (Fig. 1f). For each provenance, the maximum possible number of nodal segments and shoot tips were taken from branches sampled in February and May. When actively growing shoots from a single provenance were obtained under both conditions (mixed samples), a comparable number of explants were taken from each condition (± 4 explants).

#### In vitro culture establishment

Shoot tips and nodal segments were washed in 250 mL of tap water containing 5 mL of Mucasol (alkaline detergent; Schülke & Mayr, Norderstedt, Germany), rinsed three times with tap water, and then stored in sterile tap water for 30 min. Surface sterilization of the explants was performed by immersion for 1 min in 70% ethanol, followed by immersion for 20 min in 1% NaClO with 0.1% Tween-20 and rinsing four times for 5 min in sterile tap water under constant stirring. The surface-sterilized shoot tips and nodal segments were initially placed on M1 medium (Fig. 1f) under standard PGC conditions to promote early bud development [29]. After 4 weeks, noncontaminated explants with axillary bud development on nodal segments (Fig. 1g) or newly emerged leaves on shoot tips were transferred to medium M2 with a reduced BAP concentration. The in vitro shoots were subcultured on fresh media every 4 weeks.

#### Data acquisition

After three subculture intervals (12 weeks after in vitro culture establishment), the shoot tips and nodal segments were analysed for contamination and shoot formation. The contamination rate was calculated on the basis of the total number of explants initially used and the number lost due to fungal or bacterial contamination. For the shoot formation rate, only contamination-free explants were considered. Explants were positive for shoot formation if at least one new shoot of 1 mm in length and at least one new leaf developed under in vitro conditions. Nodal segments and shoot tips were combined for all evaluations. If several genotypes were used to establish the in vitro culture of a provenance, these were evaluated as a pool. To assess the influence of the sampling date of branches of provenance trial trees on in vitro culture establishment, explants were divided into three groups: “February”, “mixed”, and “May” samples.

### Experiment II

#### Plant material

To examine the potential positive influence of particularly young starting material on in vitro cultivability, explants from 3-week-old beech seedlings were used to establish an in vitro culture according to Barker et al. [30]. Beechnuts from Grosshansdorf, Germany, were germinated in standard plant soil under standard PGC conditions in March 2022 (beechnut year of maturity: 2021). The seedlings were pruned immediately after the primary leaves had fully expanded, approximately 3 weeks after germination. Two types of explants were tested for their impact on contamination rates and shoot formation: whole shoots (WS; N = 65), 3–4 cm in length, including cotyledonary nodes, and shoot tips (ST; N = 65), 1 cm in length, with the cotyledonary nodes removed.

#### In vitro culture establishment

WS and ST were surface sterilized as described for E I. For each type of medium, 13 WS and 13 ST were placed upright in media with varying phytohormone compositions (M0, M2, M2.1, M2.2, M3). The explants were cultivated under standard PGC conditions with a subculture interval of 4 weeks. After 8 weeks, explants grown on M2, M2.1, M2.2, or M3 were transferred to M2. The samples in M0 remained on this medium. While explants with fungal contamination were discarded, explants with bacterial contamination remained in the culture.

#### Data acquisition

Shoot formation and fungal and bacterial contamination were evaluated at each subculture interval (every 4 weeks). Explants were considered positive for shoot formation if at least one new shoot of 1 mm in length and at least one fully formed leaf emerged from the apical meristem or cotyledonary nodes. Callus formation at the base of the shoots was assessed after 8 weeks. After three subculture intervals (12 weeks after establishment), WS and ST were further evaluated for the number of shoots per explant, shoot length, chlorosis, and the formation of resting buds. The longest shoot length was recorded for explants with multiple shoots. Explants were considered positive for chlorosis or resting bud formation if at least one leaf exhibited chlorosis or if at least one resting bud had formed. The shoot formation and contamination rates were calculated on the basis of the number of shoot-forming explants relative to all initial explants. For shoot-forming explants, the median and interquartile range (IQR) of the number of shoots, shoot length, and percentage of chlorotic explants and explants with resting buds were calculated. The median was chosen for shoot length and shoot number because of its robustness against outliers and the nonnormal distribution of the data.

### Experiment III

#### Plant material

For the optimization of the protocol used in E II, beechnuts from the commercially available provenance “81009” (Harz, Weser- und Hessisches Bergland, Germany; year of maturity: 2022) were obtained from the State Forestry Department of Brandenburg (Waldsieversdorf, Germany). Because these seeds presented a low germination rate, seeds from the same provenance were additionally derived from the State Forestry Department of Lower Saxony (Oerrel, Germany). Seeds were stored at -8 °C until germination was initiated in soil under standard PGC conditions between June and September 2023. The preparation of seedlings was carried out following the procedures outlined in E II. To reduce the risk of contamination and the formation of resting buds, only ST were utilized.

#### In vitro culture establishment

The sterilization protocol used in E I and E II was modified: After washing with the Mucasol mixture, the ST were rinsed thoroughly under running tap water. Sterilization was carried out using 5% NaClO solution for 5 min. To test the ability of antibiotics to control bacterial contamination, the shoot tips were then washed three times for 5 min in sterile tap water either with or without antibiotics (500 mg/L cefotaxime, 50 mg/L gentamicin, 50 mg/L streptomycin). Afterwards, the ST explants were placed upright in phytohormone-free medium (M0), as shoot formation was highest on M0 in E II until week 8, or in M0-AB if the explants were previously treated with antibiotics. All explants were cultivated under standard PGC conditions. After 6 weeks, fungal contamination-free explants were transferred to phytohormone-supplemented M2+ medium for multiple shoot induction and to address the chlorosis and resting bud formation observed in E II.

To test the growth of *F. sylvatica *in vitro plantlets under autotrophic conditions, explants were evenly divided into M2+ with sucrose and M2+ without sucrose (M2+ woS). The choice of M2 + woS was based on positive results from previous tests with plantlets of genotypes from E I and E II, which showed sufficient growth on this medium. The subculture interval was extended to 6 weeks on the basis of the successful culture of genotypes from earlier experiments with the same medium without signs of chlorosis or loss of shoot vitality.

#### Data acquisition

After two subculture intervals (12 weeks after establishment), ST were evaluated for fungal and bacterial contamination, shoot formation, shoot length, and number of shoots per explant as described for E II.

### Statistical analysis and graphics

Statistical tests in E I–E III were performed, and graphics were created via the open‐access statistical computing software R version 4.3.1 (https://www.R-project.org) with RStudio version 2023.6.1.524 (http://www.posit.co). Statistical analysis of nominal-scaled data was subjected to hypothesis testing via Pearson’s chi-squared independence test to assess the stochastic independence between two discrete random variables. In cases where the assumption of the chi-squared test was not met due to low expected cell frequencies (ñij < 5) in the contingency tables, a transition was made to Fisher’s exact test. After obtaining a significant result from the Pearson’s chi-squared independence test or Fisher’s exact test, pairwise tests were conducted to further explore specific differences among categories with the pairwise_prop_test function from the R package Rstatix or the Fisher.multcomp function from the R package RVAideMemoire, respectively, with the p.adjustment method Benjamini & Hochberg (false discovery rate).

Statistical analyses of the metrically scaled data were subjected to the assumption of parametric tests. The normality of the distribution of the data and residuals was assessed via the Shapiro‒Wilk test, whereas the homogeneity of variance was examined via Levene’s test. Due to irregularities in meeting these assumptions, statistical analyses of all the metrically scaled data were performed with nonparametric tests. Differences between two independent samples were investigated via the Mann‒Whitney U test from the R package exactRankTest. Statistical analyses involving three or more independent groups were conducted via the Kruskal‒Wallis test, followed by multiple median comparisons via Dunn’s test for multiple comparisons (post hoc test) with the p.adjustment method Benjamini & Hochberg (false discovery rate). To minimize bias, only sample groups with N ≥ 10 were included in the analysis.

To evaluate the impact of independent variables (provenance, tree age, and sampling date) on shoot formation and contamination rates in E I, a logistic regression model was employed. To minimize bias, only sample groups with N ≥ 10 were included in the analysis. Unlike linear regression, logistic regression does not assume normality of independent variables or residuals, which is appropriate for binary outcomes (i.e., shoot formation and contamination). This model accommodates both categorical (e.g., sampling date, provenance) and continuous (e.g., age) predictors, making it suitable for mixed datasets. The influence of the sampling date (predictor) on shoot formation or the contamination (dependent variable) was analysed via the glm() function from the R stats package, with the family set to binomial. The model formula was structured as dependent_factor ~ predictor. To investigate the effects of provenance and tree age (predictors) on the shoot formation rate, logistic regression with Firth correction was applied via the logistf() function from the R logistf package, with the family set to binomial. Firth correction was used to address issues related to perfect separation, providing more robust estimates of regression coefficients. This penalized maximum likelihood approach is particularly useful when dealing with small sample sizes or multicollinearity, helping to stabilize estimates and improve inference reliability. The reference category for provenance was adjusted via the relevel() function. Considering the effect of sampling date, provenances 13, 10, and 19 were chosen as the reference groups, as they showed the lowest shoot formation among all provenances and February samples, among the May samples, and among the mixed samples, respectively. To assess interactions between multiple predictors, extended models incorporating two predictors were tested. The model formula was structured as dependent_factor ~ predictor1 + predictor2. When the maximum number of iterations for the full model was exceeded, leading to convergence issues, the number of iterations increased (logistf.control(maxit = 1000)). If convergence problems persisted, the maximum number of iterations for the penalized likelihood (PL) confidence intervals was further increased (logistpl.control (maxit = 50000)).

The individual effects of the predictors and the overall model strength were validated via the Wald test, implemented via the wald.test() function from the R lmtest package. To assess the performance of the logistic regression model, the predicted probabilities for each observation were calculated via the predict() function from the stats package. A confusion matrix was then created using ifelse(), comparing actual and predicted classes on the basis of a defined threshold. The matrix provided true positives (TPs), false positives (FPs), true negatives (TNs), and false negatives (FNs). The optimal threshold was determined by plotting an ROC curve via the roc() function from the pROC package, with the threshold selected on the basis of the maximum sum of sensitivity and specificity. The area under the curve (AUC) was calculated with auc() from the package pROC to evaluate the model's discriminative ability, where an AUC of 1 indicates perfect classification and 0.5 suggests random guessing.

Graphics were created via the R package ggplotII for dot plots. The R package tidyr was also used for line charts. For maps ggplotII, sf, mapsF, and rnaturalearth were used. The distribution area of *F. sylvatica* was adopted from Caudullo et al. [53].

### Rooting and *ex vitro* acclimatization of microcuttings

To induce root growth in vitro, 10 microcuttings from different seedling-derived genotypes maintained on M2+ medium under standard PGC conditions were used. Shoots that were at least 1 cm in length were freshly cut at the base before being dipped in a 5 mM IBA solution for 1 min. Then, the shoots were cultivated upright in medium M0+ (M0 supplemented with 50 µM FeNaEDTA and 400 µM MgSO_4_) under standard PGC conditions for 3 weeks. For acclimatization to *ex vitro* conditions, rooted shoots were placed in either 1:1 perlite–soil or expanded clay–soil mixtures. The vessels were covered and placed in a PGC under standard conditions. The humidity was gradually adjusted to 60% over 3 weeks by stepwise removal of the cover.

## Results

### Experiment I

#### Bud break

For 18 of the 19 provenances sampled in February, bud break was initiated under both PGC (Fig. 1c) and GH (Fig. 1d) conditions. Provenance 9 (Westerwald, DE) exhibited bud break only under PGC conditions, whereas in seven provenances, bud break occurred exclusively under GH conditions. Eight provenances failed to initiate bud break under both conditions. In May, all 17 sampled provenances exhibited successful bud break under GH conditions. Actively growing shoots were obtained from all previously unsuccessful provenances, except for provenance 22 (Cer, RS). An insufficient tree size prevented a second sampling of this provenance. For the same reason, it was only possible to harvest provenances 1, 13, 15, and 20 in February, but these samples showed successful bud break. In addition, provenances 4, 5, 8, and 10 were harvested in May to maintain a large total quantity of provenances.

#### Sampling date

A total of 691 nodal segments and shoot tips from 22 of the 23 provenances sampled in February and/or May were used for in vitro culture establishment (Table 2). Among the 691 explants, 360 were lost to contamination in the further stage (52%). The contamination rates during subsequent cultivation varied between provenances from 0 to 100%. All May samples from provenance 11 (ES; N = 21) were lost because of contamination, while no losses were observed in February samples from provenance 20 (PT; N = 27). The contamination rate and shoot formation was evaluated in relation to the harvest time in February or May to identify possible correlations. Three groups are distinguished here: (i) provenances that sprouted in February, (ii) those sprouted in May, and (iii) those sprouted in both February and May, referred to as ‘mixed’. The last group was considered together because their explants were not cultivated separately after in vitro establishment. Analyses via Pearson’s chi-squared test demonstrated that the sampling date significantly affected both the contamination rate (χ^2^(2) = 67.45, p < 0.001) and shoot formation (χ^2^(2) = 8.069, p = 0.018). Subsequent pairwise Pearson’s chi-squared tests indicated that the contamination rates among all groups (February, mixed, and May samples) were significantly different (p ≤ 0.001, Table 3). February samples (27%) produced less contamination than May samples (69%).

**Table 3:** Effects of sampling date on contamination and shoot formation rates

Sampling date	N provenances	N genotypes	Σ samples %	Contamination %	Shoot formation %
Feb	5	10	137	27^a^	5^a^
Mixed	5	13	271	48^b^	18^b^
May	12	18	283	69^c^	16^ab^

Branches were collected in February (Feb) and/or May. Among the Feb samples, 244 samples were derived from bud break under GH conditions, and 29 were derived from bud break under PGC conditions. For the mixed samples (Feb/May), the same number of samples (± 4 explants) were derived from the Feb or May samples. Shoot formation rates were calculated after the exclusion of contaminated explants. Values with different letters are significantly different according to the pairwise Pearson’s chi-squared test with p < 0.05

**Fig. 2:**
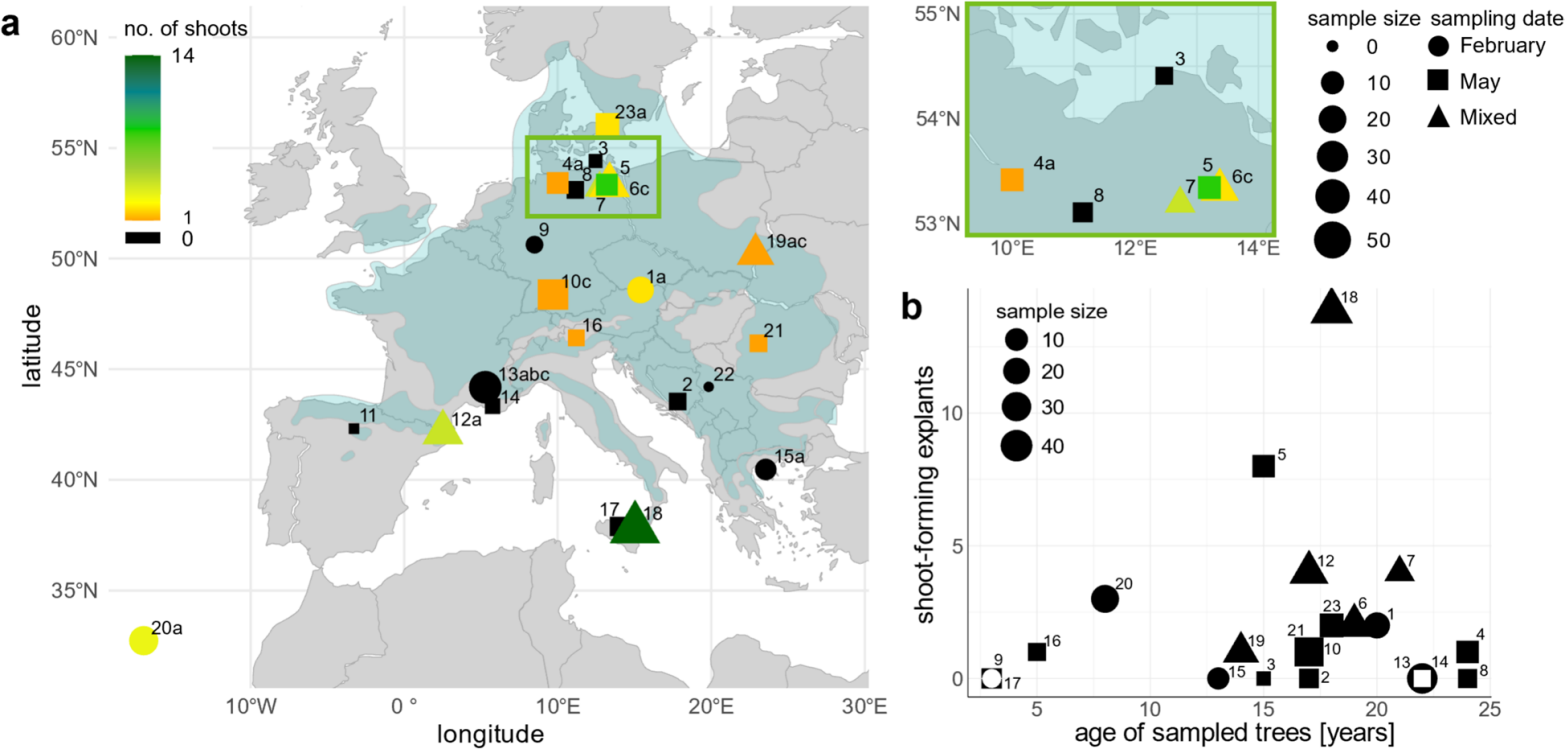
Shoot formation of beech explants taken from actively growing shoots of provenance trial trees. The evaluation was performed after 12 weeks of in vitro cultivation. Branches were sampled in February (marked by circles), May (squares), or both February and May (triangles). The provenances are labelled by number according to Table 2. **a** Shoot-forming explants depending on the provenance. The colour indicates the number of shoot-forming explants. Provenances without shoot formation are coloured black. Shoot formation was successful in 13 of the 23 provenances sampled (57%). Provenance 11 was lost due to contamination, whereas bud break was unsuccessful in provenance 22. The green box highlights the area where six provenances are shown on a larger scale for spatial distinction. The shoot formation rates of provenances 5, 18, and 7 are significantly higher than those of the provenances labeled with the corresponding letters (**a**, **b**, or **c**, respectively), according to pairwise Fisher's exact test (p < 0.05). Provenances with a sample size N < 10, after excluding contaminated explants, were not included in the statistical analysis. The distribution area of *F. sylvatica* is highlighted in blue. **b** Shoot-forming explants based on the age of the sampled trees. Provenances 9 and 17 as well as 13 and 14 overlap in this graph

For the remaining non-contaminated 331 explants, shoot formation was recorded. A total of 44 explants produced shoots (Table 2), yielding a shoot formation rate of 13.3% (6.4% of total explants) after three subculture intervals (12 weeks, Fig. 2a). Statistical analyses demonstrated a significant effect of sampling date on shoot formation (χ^2^(2) = 6.37, p = 0.041). However, based on our data and considering all initial explants, differences in shoot formation rates were observed for February, May, and mixed samples (May: 5%; February: 4%, Mixed: 9%), but these differences were not significant (p ≥ 0.066). After excluding contaminated explants, the February samples presented a significantly lower shoot formation rate (5%) than the mixed samples did (18%, p = 0.026), whereas the May samples presented an intermediate rate (16%, p > 0.05; Table 3). Logistic regression confirmed the effect of sampling date on shoot formation (χ^2^ = 7.197, p = 0.027). Compared with the February samples, both the mixed (coefficient = 1.358, p = 0.008) and the May samples (coefficient = 1.210, p = 0.026) positively influenced shoot formation and, thus, presented a greater shoot formation rate.

#### Provenances

Among the 23 initial provenances, 13 successfully formed shoots in vitro (57%; Fig. 1h), whereas shoot formation failed in nine provenances (Fig. 2a). Most shoot formation was obtained from provenance 18 (IT) in 14 samples out of 72 explants. Provenance 5 (DE) obtained the highest shoot formation rate (80%). The provenances 16 (IT) and 21 (RO) formed one shoot each, even though their sample sizes were small, with only 3 and 4 explants, respectively. Due to the small sample sizes, these two provenances were not considered in subsequent statistical analyses despite their high shoot formation rates.

Without considering contaminated samples, 13 provenances (with sample sizes ≥ 10) remained for analysing provenance effects on shoot formation (1, 4–7, 10, 12, 13, 15, 18–20, 23). Fisher’s exact test indicated significant variation across provenances (p ≤ 0.001; Fig. 2a). Provenance 13 (DE), sampled in February, exhibited the lowest shoot formation of all provenances (0%; N = 38). Compared with provenance 13 (FR), the shoot formation rates of provenances 5 (DE; May samples), 7 (DE; mixed samples), and 18 (IT; mixed samples) were significantly higher. Provenance 19 had the lowest shoot formation rate among the mixed provenances (4%, N = 24), which was significantly lower than that from those of provenances 5 and 18. Furthermore, provenance 18 displayed a significantly higher shoot formation rate than did provenance 10 (DE), which exhibited the lowest shoot formation rate among the May-sampled provenances (3%; N = 31).Firth’s logistic regression was employed to further analyse the effect size of provenance on shoot formation. The influence of provenance on shoot formation was validated (Wald test, χ^2^ = 37.79, p < 0.001). Within this analysis, provenance 5 (DE) had the strongest positive effect on shoot formation (coefficients = 4.236 and 3.975, respectively; p < 0.001) compared with provenances 10 and 19 (PL), followed by provenances 7 (DE; coefficients = 2.501 and 2.241, p = 0.009 and 0.018) and 18 (IT; coefficients = 2.302 and 2.041, p = 0.001 and 0.006). When provenance 13 (FR) was used as the reference group, additional provenances (20 (PT), 23 (SE), and 12 (ES)) had positive effects (p ≤ 0.05), although these effects were less pronounced. Provenance 20 was the only one derived from February samples.

#### Statistical limitations

The age of trees with successful shoot formation in vitro ranged from five years (provenance 16, IT) to 24 years (provenance 4, DE). Both provenances with three-year-old trees failed to produce shoots (provenance 9 (DE), N = 7; provenance 17 (IT), N = 32; Fig. 2b). After excluding five-year-old trees from provenance 16 (IT) due to the small sample size (N = 3), the effect of tree age on shoot formation was analysed. Fisher’s exact test indicated significant variation across age groups (p ≤ 0.001). Subsequent pairwise comparisons highlighted significantly greater shoot formation for trees aged 15, 18, and 21 years than for those in other age groups (Additional file 2). Those age groups mainly consisted of provenances 5, 7, or 18, which previously presented improved shoot formation. Analysing age as a continuous variable in a Firth-corrected logistic model with or without the use of the sampling date as a second predictor indicated a marginal, nonsignificant negative effect of increasing age on shoot formation (coefficient = − 0.024, p = 0.580). The logistic regression model’s validity was limited due to iteration constraints when both provenance and sampling date or provenance and age were included as predictors. The data is not sufficient to include the effect of age or sampling date on shoot formation in vitro during analysing provenance-specific differences.

### Experiment II

**Fig. 3:**
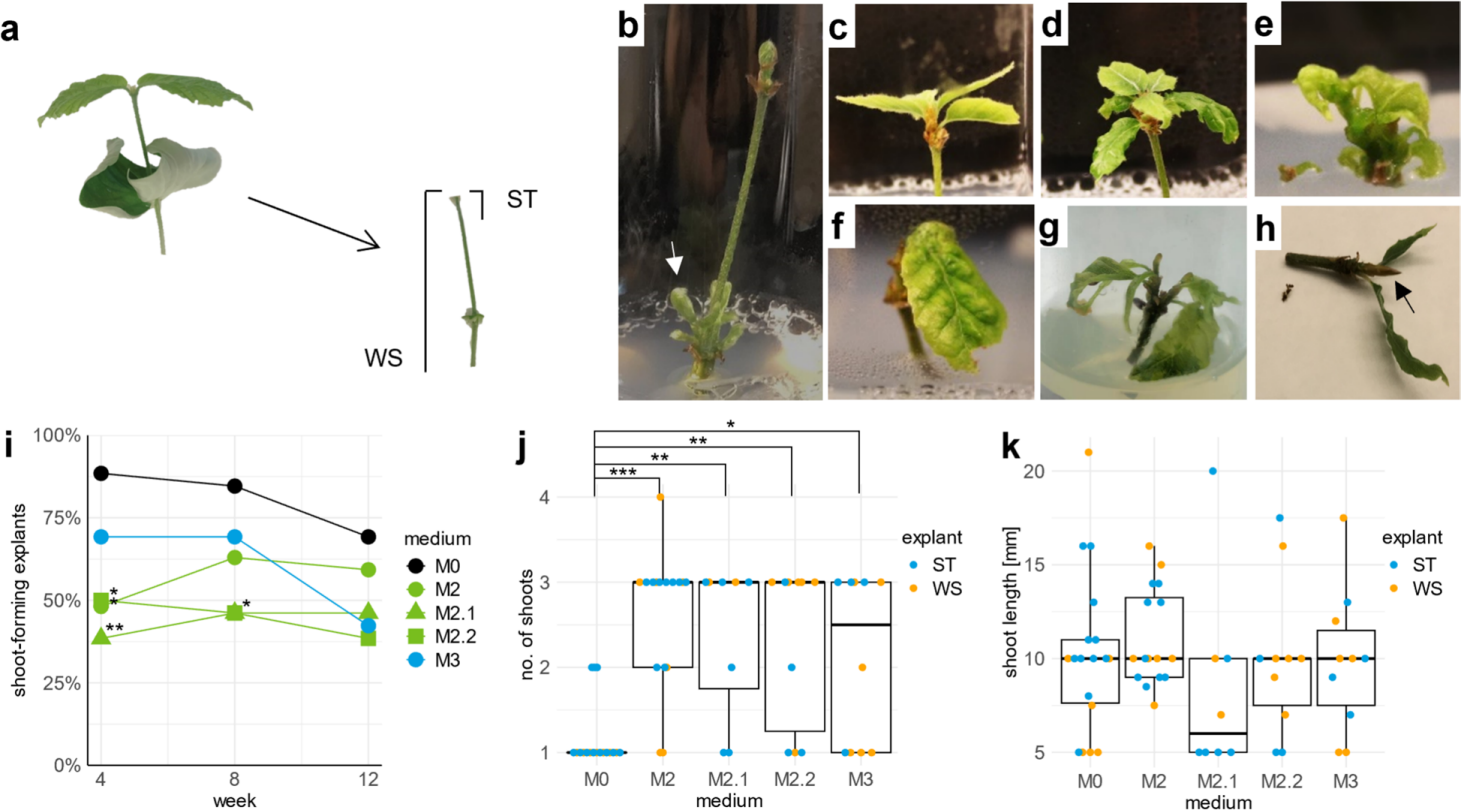
In vitro culture establishment of beech via whole shoots and shoot tips of seedlings. **a** Seeds collected from Grosshansdorf (DE) were germinated in March in a plant growth chamber in cultivation soil. Once the first true leaf pair fully expanded, the leaves were removed, and the shoots were sterilized. The plants were divided into whole shoots (WS) and shoot tips (ST) and cultured on WPM with 2% sucrose and 0.65% agar, pH 5.6, supplemented with no hormones (M0) or different hormone concentrations (M2, M2.1, M2.2, and M3). **b** Bud break on cotyledonary nodes (white arrow) after 4 weeks of culture. **c–f** Explant phenotypes after 8 weeks **c** on M0 and **d** on M2. Some explants on M2.2 and M3 showed **e** hyperhydricity or **f** chlorosis. **g** Shoot multiplication occurred via axillary branching after 12 weeks. **h** Explant with resting buds (black arrow) after 12 weeks. **i–k** After 8 weeks, explants on M2, M2.1, M2.2, and M3 were transferred to M2, whereas M0 explants remained on M0. The groups were named according to their initial media. Analyses based on the media used were conducted for **i** percentage of vital shoot-forming explants after 4, 8, and 12 weeks (ST and WS were pooled). Box plots show **j** the median number of shoots per explant and **k** shoot length after 12 weeks. Each dot represents data from a single explant, with ST and WS differentiated by color. Significant differences from medium M0 were determined by **i** Pearson’s Chi-squared or **j, k** Dunn’s test (*p < 0.05, **p ≤ 0.01, ***p ≤ 0.001)

As a particularly young starting material for in vitro establishment, both whole shoots (WS) and shoot tips (ST) of beech seedlings were used (Fig. 3a), the latter with the intention of using even less tissue to keep the contamination rate low. In total, 130 WS and ST from seedlings were introduced into in vitro culture media. Considering all explant types 12 weeks after in vitro establishment, bacterial contamination was observed in 43% of the explants (Table 4), and 16% of the explants were lost due to fungal contamination. No significant differences in bacterial (Pearson’s chi-squared test, χ^2^(4) = 6.55, p = 0.162) or fungal (Fisher’s exact test, p = 1) contamination rates were found between the different culture media. However, independent of the medium, both bacterial and fungal contamination were significantly greater in WS than in ST.

**Table 4:** Effect of the explant type on in vitro performance of beech explants obtained from seedlings

Explant type	Fungal contamination		Bacterial contamination		Resting buds	
	%	p value	%	p value	%	p value
Pooled	16	–	43	–	17	–
Whole shoots	31	< 0.001	55	0.011	37	< 0.001
Shoot tips	1		32		3	

The evaluation of seedling-derived explants was performed after 12 weeks of in vitro culture. The shoot tips (ST) and whole shoots (WS) were either separately evaluated or combined (pooled). Differences between ST and WS were analysed using pairwise Pearson’s chi-squared test

Meristem sprouting was recorded every 4 weeks on the basis of shoot formation on the explants. After the first 4 weeks, 38 ST and 39 WS presented shoot formation. Over the entire 12-week observation period, 18 ST and 25 WS never sprouted, including explants that were lost to fungal contamination. In addition to shoot formation on the apical meristem, bud break on the cotyledonary nodes was observed, but only on phytohormone-supplemented media (Fig. 3b).

In addition to shoots, leaves also formed on the sprouted explants. Explants grown on the hormone-free medium M0 produced thin, flat leaves, whereas those on phytohormone-containing media developed thicker, wavier leaves, resembling the morphology of *ex vitro* beech leaves (Fig. 3c, d).

#### Four weeks of cultivation

After 4 weeks and across all explants, the shoot formation rate on the hormone-free medium M0 was significantly higher than that on M2 (p = 0.022), M2.1 (p = 0.005), and M2.2 (p = 0.024) (Pearson’s chi-squared test, χ^2^(4) = 17.14, p = 0.002). In medium M2, the shoot formation rate of WS (70%) was significantly higher than that of ST (23%) (Fisher’s exact test, p = 0.045). Furthermore, no significant differences were found between the ST and WS explant types on the different media, e.g., in terms of shoot number or shoot length.

Even in the subsequent test cycles in E II, despite differences in the shoot formation rate on M2 after 4 weeks, no significant deviations between WS and ST considering shoot formation rate, shoot number, and shoot length were found according to Pearson’s chi-squared or Kruskal‒Wallis test (see Additional file 3 for data regarding WS and ST).

#### Eight weeks of cultivation

After 8 weeks, the shoot formation rate on M0 (85%) was still significantly higher than on M2.1 and M2.2 (both media 46%, p = 0.044), whereas the shoot formation rates on M2 (63%) and M3 (69%) were intermediate. Callus formation at the shoot base was observed on M2.2 and M3 (35% and 26%, respectively). On these two media, unfavourable effects, such as hyperhydricity (Fig. 3e) and slight chlorosis (Fig. 3f), were observed in some explants. Additionally, browning occurred, resulting in a decrease in shoot-forming explants between weeks 4 and 8 on media M2.2 and M0.

Since shoot formation was lowest on M2.1 and M2.2 and chlorosis was observed on M3 but not M2, all WS and ST explants cultivated on phytohormone-containing media were transferred to M2 after week 8 to avoid loss of genotypes. Although the explants on phytohormone-containing media were all grown on M2 medium, the explants were analysed separately according to their original media to avoid misleading data interpretation.

#### Twelve weeks of cultivation

As a result of browning of in vitro shoots, the number of shoot-forming explants further decreased between weeks 8 and 12 for all media except explants initially cultured on M2.1 (Fig. 3i). By the third subculture interval (12 weeks), the shoot formation rates were 38% for M2.2 and 69% for M0, although these differences were not statistically significant. The highest shoot formation rate (85%) was observed in M0 when ST was used.

As a final analysis after 12 weeks, the multiplication rate of the explants was determined on the basis of the number of shoots per explant since each shoot can be found as an independent explant. Explants on M0 produced a median of one shoot per explant (IQR = 0). Within the explants transferred to medium M2, between 2.5 (IQR = 2.0; M3) and 3.0 (IQR = 1.0; M2, M2.1, M2.2) shoots via axillary branching were obtained (Fig. 3g). A Kruskal‒Wallis test showed significant differences in shoot number among the media (χ^2^(4) = 22.7, p ≤ 0.001), with Dunn’s post hoc test confirming that the culture on M0 differed significantly from all other phytohormone-containing cultures (p ≤ 0.032; Fig. 3j). No significant differences in shoot number were observed within the group of explants cultured on phytohormone-containing media.

Across all media and explant types, the shoot lengths varied from 6.0 mm (± 5.0 mm) for cultures originally on M2.1 to 10.0 mm (± 2.5 mm) for those originally grown on M2.2 (Fig. 3k), but these differences were not significant (Kruskal‒Wallis test, χ^2^(4) = 4.23, p = 0.38). Overall, 51% of the explants (67 genotypes) developed in vitro shoots with a median length of 10.0 mm (± 4.3 mm).

The viability of the grown shoots was assessed via chlorosis and resting bud formation. Although the plants were transferred to M2 in week 8, chlorosis was observed on all the shoots initially cultured on M2.2 and M3, whereas significantly fewer shoots on M0 (33%), M2 (25%), and M2.1 (17%) presented signs of chlorosis (Pearson’s chi-squared test, p ≤ 0.001). Growth arrest was indicated by the formation of resting buds (Fig. 3h) in 17% of all explants. WS had a significantly greater percentage of resting buds (37%) than did ST (3%) (Pearson’s chi-squared test, p ≤ 0.001), but the differences between media were not significant (p ≥ 0.441).

### Experiment III

**Fig. 4:**
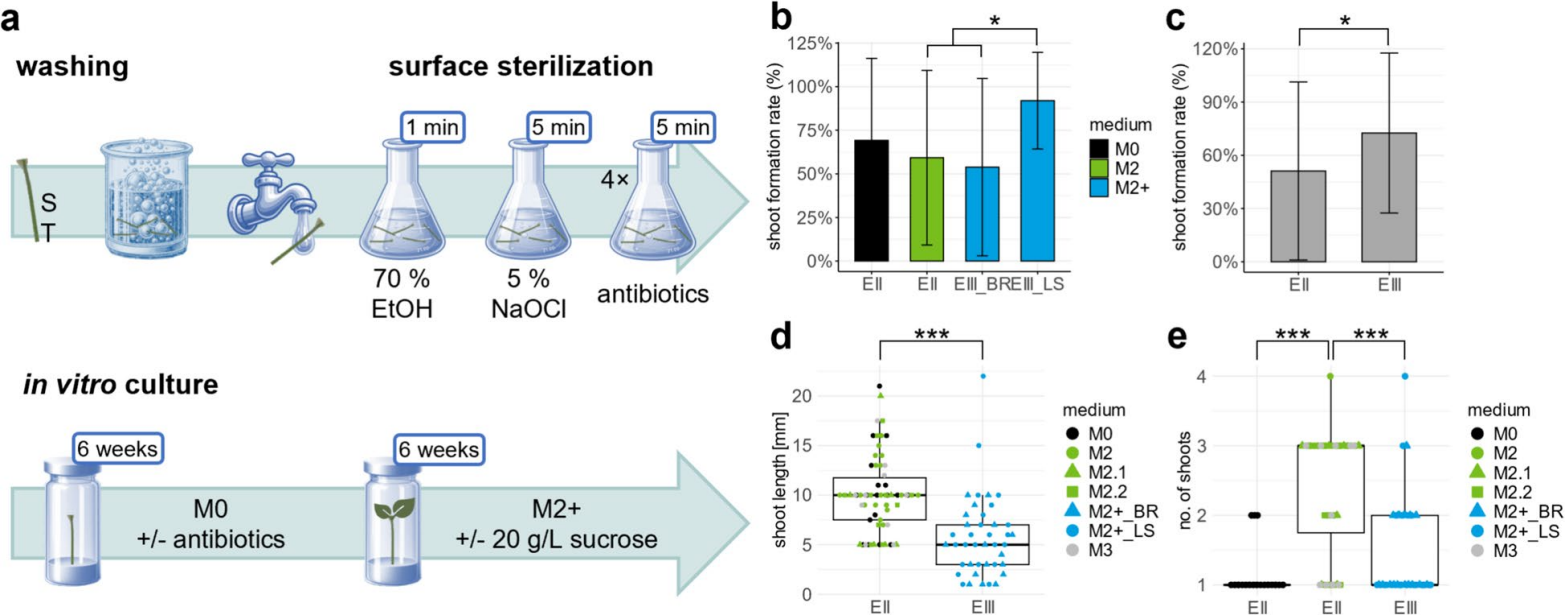
In vitro culture establishment using shoot tips of seedlings in combination with antibiotics. **a** In Experiment III (E III), shoot tips (ST) were prepared after full expansion of the first leaf pair. ST were washed with an alkaline cleaning agent, rinsed under tap water, and stored in sterile water for 30 min. Surface sterilization involved 70% EtOH (1 min) and 5% NaOCl (5 min), followed by rinsing in sterile water with or without antibiotics. Antibiotic-treated ST were cultured on medium M0 supplemented with antibiotics. After 6 weeks, ST were transferred to M2+ with or without 2% sucrose. After 12 weeks, the ST were evaluated for **b** shoot formation rate based on seed supplier (State Forestry Departments in Brandenburg (M2+ _BR) or Lower Saxony (M2+ _LS)), as well as for **c** shoot formation rate, **d** shoot length, and **e** number of shoots per explant, all pooled for E III on medium M2+ /M2+ woS. The results from M2+ and M2+ woS were compared to those from Experiment II (E II) with seedlings on M0 or M2 for 12 weeks or on M2.1, M2.2, or M3 for 8 weeks plus 4 weeks on M2. The groups were named after their original media in E II. Significant differences were assessed via **b, c** Pearson’s Chi-squared test, **d** Mann‒Whitney U test, or **e** Dunn’s test, with *p < 0.05 and ***p ≤ 0.001

Based on the findings from E I and E II, ST from beech seedlings were introduced into in vitro culture. The protocol included a modified sterilisation procedure and the addition of antibiotics to the medium (Fig. 4a). The use of antibiotics during in vitro culture establishment significantly reduced bacterial contamination in ST explants from 41% (samples without antibiotics) to 0% (with antibiotics, Table 5). No significant difference in fungal contamination was observed. No critical influence was observed, with rates of 0% and 3%, respectively, in the two sample groups.

**Table 5:** Effects of antibiotic treatment on in vitro establishment

Antibiotics	N	Fungal contamination		Bacterial contamination		Shoot formation rate		Shoots per explant (median ± IQR)		Shoot length (median ± IQR)	
		%	p value	%	p value	%	p value	N	p value	[mm]	p value
Not added	17	0	1.000	41	< 0.001	94	0.019	1.0 (± 1.00)	0.608	6.0 (± 4.75)	0.231
Added	34	3		0		62		1.0 (± 1.00)		5.0 (± 4.00)	

Cefotaxime (500 mg/L), gentamicin (50 mg/L), and streptomycin (50 mg/L) were either added or not added during sterilization and in the medium. The evaluation was performed after 12 weeks of in vitro culture. The sample size (N) of shoot tips grown on propagation media supplemented with or without 2% sucrose was ± 1. Differences between “not added” and “added” were analyzed by Fisher’s exact test for contamination and shoot formation rates or the Mann‒Whitney U test for the number of shoots and length

IQR, interquartile range

Antibiotic treatment had no significant effect on the number of shoots formed per explant or the length of the shoots that developed 12 weeks after culture establishment. However, the shoot formation rate was significantly lower in ST treated with antibiotics than in ST without antibiotics (Table 5).

All explants not treated with antibiotics were derived from seeds from Lower Saxony. Explants that received the antibiotic treatment were a mix of both seed suppliers. With respect to the different seed suppliers, no significant difference in the shoot formation rate was found between ST from Lower Saxony treated with (88%) or without antibiotics (94%) according to pairwise Fisher’s exact test (p = 1). Considering all explants, shoot formation was significantly lower in ST from Brandenburg (54%) than in those from Lower Saxony (92%; Fig. 4b). However, the beechnut germination rates in the soil prior to the ST preparation were significantly lower for seeds from Brandenburg (14%) than for those from Lower Saxony (71%) (p < 0.001).

For further cultivation, media supplemented with sucrose (M2+) were tested for comparison with media without sucrose (M2+ woS). No significant differences were detected in the shoot formation rate, shoot number, or shoot length between explants grown on M2+ or M2+ woS during the second subculture interval (Table 6).

**Table 6:** Effects of sucrose in propagation media on contamination and shoot formation in vitro

Medium	N	Fungal contamination %	Bacterial contamination %	Shoot formation rate %	Shoots per explant (median ± IQR)	Shoot length [mm] (median ± IQR)
M2 +	26	0	8	73	1.0 (± 1.00)	5.5 (± 3.25)
M2 + woS	25	4	20	72	1.0 (± 0.50)	5.0 (± 5.50)

The evaluation was performed 12 weeks after the introduction into in vitro conditions. The sample sizes (N) of shoot tips from Lower Saxony or Brandenburg treated with or without antibiotics were ± 1 between the groups grown on M2 + media supplemented with or without 2% sucrose (M2 + woS). According to Fisher’s exact test, no significant differences were identified for any of the parameters. IQR – interquartile range

### Media optimization

In E III, the M2 medium was supplemented with FeNaEDTA and MgSO_4_ (M2+) to prevent the loss of in vitro shoots that formed during establishment. A comparison of the shoot formation rates after 12 weeks of in vitro culture across the media used in E II and E III demonstrated a significant effect of the media on in vitro shoot formation (Pearson’s chi-squared test, χ^2^(5) = 13.87, p = 0.016). However, no significant differences were observed between the individual media. Significant differences were only identified when the shoot formation rates of M2+ , which were categorized based on the seed supplier, were compared with those of media M0 and M2 used in E II (Fig. 4b). The shoot formation rate of M2 + for explants derived from Lower Saxony seeds (92%) was significantly greater than that of M2 (69%). A comparison between E II and E III without considering individual media indicated significant differences: the shoot formation rate in E II with explants from Grosshansdorf, Germany, cultivated on WPM-based M0, M2, M2.1, M2.2, or M3 (51%) was significantly lower than that in E III with explants from Lower Saxony and Brandenburg cultivated on media M2+ and M2+ woS based on WPM enriched in FeNaEDTA and MgSO_4_ (73%; Fig. 4c). Conversely, shoot length was significantly reduced from a median of 10 mm (IQR: 4.0 mm) in E II to 5.0 mm (IQR: 4.0 mm) in E III (Fig. 4d), during which the subculture interval was extended to 6 weeks.

In E III, explants were cultivated on M0 during the first 6 weeks of in vitro culture before being transferred to phytohormone-containing media, which are known to be needed for multiple shoot induction in E II. Comparable with explants cultured on M0 media in E II, one shoot (IQR = 1.0) formed from one explant in E III (Fig. 4e). The number of shoots was significantly greater in explants cultured on M2, M2.1, and M2.2 (median = 3.0, IQR = 1.00–1.75) than in those cultured on M2+ (Dunn’s test, p ≤ 0.021). When the data of phytohormone-containing media from E II were pooled, the number of shoots significantly decreased on M2+ in E III (Fig. 4e).

### Rooting and acclimatization of microcuttings

**Fig. 5:**
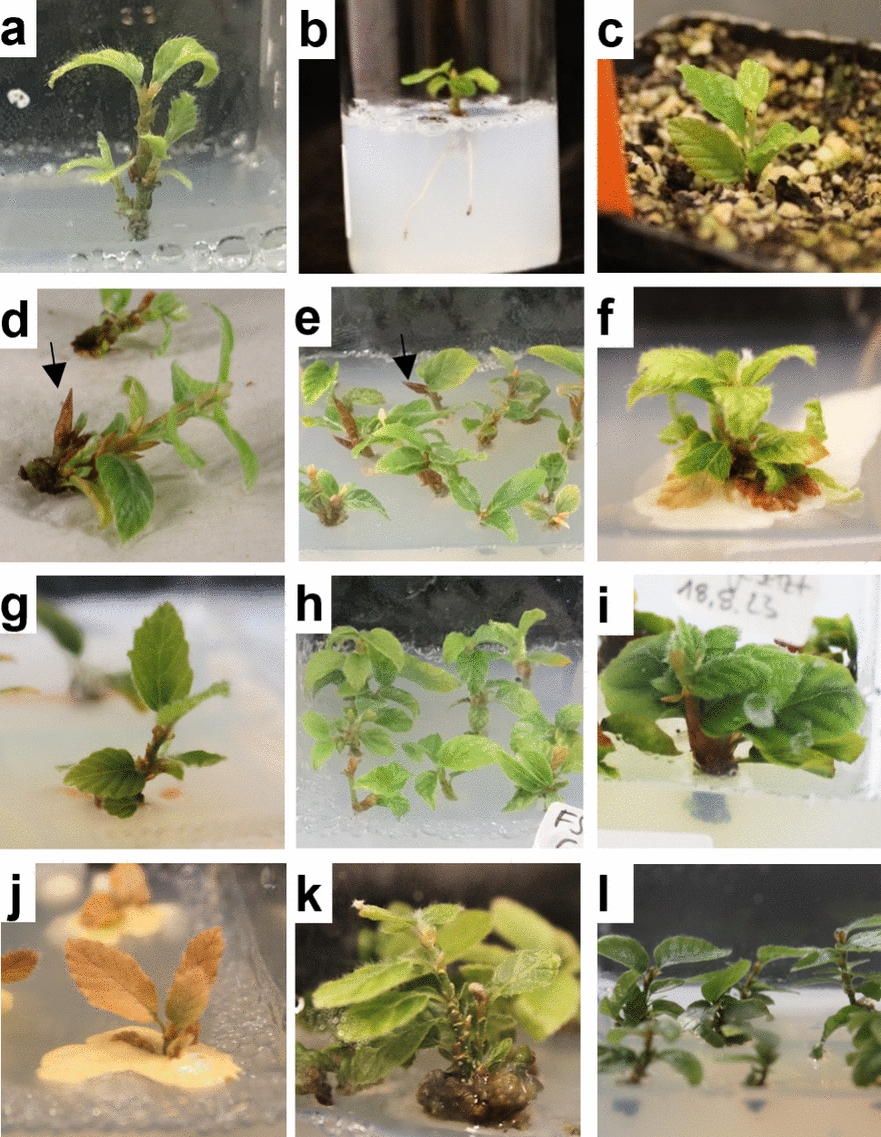
Beech microcuttings on different media compositions. **a** Shoot multiplication on medium M2 + occurred mainly via axillary branching. **b** Rooted microcutting 3 weeks after root induction by an IBA dip. **c** Rooted microcutting 5 weeks after transfer to a 1:1 perlite–soil mixture. **d, e** Formation of resting buds (arrow) and weak shoot elongation was achieved on medium M2 + containing 20 g/L sucrose. **f** Some genotypes showed bacterial overgrowth. **g** Switching to sugar-free medium M2 + woS reduced bacterial growth and **h, i** promoted outgrowth of buds previously formed. **j** Using 20 g/L glucose in medium M2 + G led to death of shoots caused by bacterial overgrowth in genotypes previously showing bacteria. **k** The other genotypes showed callus formation and shoot multiplication via adventitious shoot formation. **l** Reducing the light intensity from 70 to 30 µmol m^−2^ s^−1^ improved the overall vitality of shoots grown on M2+ G, as indicated by deeper green leaves

Root induction on microcuttings from seedling-derived genotypes (Fig. 5a) was successful in five of the ten microcuttings. One to two roots developed within three weeks after IBA dip (Fig. 5b). Prolonged shoot elongation after acclimatization was successful in two of the 4 rooted in vitro shoots placed in a 1:1 perlite:soil or a 1:1 expanded clay:soil mixture (Fig. 5c).

### Micropropagation routine and carbohydrate source testing

Over the course of in vitro culture, several genotypes from both provenance trial trees and seedlings (E I–III) presented a decline in the proliferative capacity on M2+ . This decline was characterized by chlorosis, weak shoot growth, and the formation of resting buds (Fig. 5d, e). Switching to M2+ woS resulted in a noticeable reduction in chlorosis. Additionally, in cultures with excessive bacterial growth on medium M2+ , bacterial overgrowth was suppressed by cultivation on M2 + woS (Fig. 5f, g).

Shoot formation occurred primarily through lateral branching or axillary shoot development in both M2+ and M2+ woS plants (Fig. 5a, e, h). Additionally, bud formation at the shoot base was observed on M2+ , although these buds often failed to grow further (Fig. 5d, e). However, transfer to M2+ woS successfully promoted the sprouting of resting buds (Fig. 5i). Despite these adjustments, shoot elongation remained limited in both M2+ and M2+ woS (Fig. 5e, h).

In contrast, introducing glucose in the medium (M2+ G) resulted in extensive bacterial overgrowth in genotypes previously susceptible to bacterial contamination, leading to shoot death (Fig. 5j). However, genotypes free from bacterial contamination demonstrated enhanced growth on M2+ G, with longer shoots and more callus formation at the shoot base than those cultured on M2+ or M2+ woS. In these genotypes, shoot multiplication was not delayed by the formation of resting buds as observed on M2 + . The newly formed buds underwent bud break directly, developing into new shoots (Fig. 5k). Improved shoot vitality, indicated by more vigorous growth and deeper green leaves, was observed when the light intensity during culture on M2+ G was reduced to 30 µmol m⁻^2^ s⁻^1^ (Fig. 5l).

Two years after in vitro culture establishment in E I, only provenance 19 (PL, 4%) showed vital growth on M2+ G. From E II, 10 out of 67 genotypes from Grosshansdorf (DE, 8%) showed vital growth after two years, all performing best on M2+ woS. Massive bacterial growth and shoot browning were observed on M2 + G but not on M2+ or M2+ woS (Fig. 5d–f). In E III, 15 of the 37 genotypes from provenance 81,009 (DE) with successful shoot formation grew after approximately 6 months, representing 27% of the initial 51 seedlings. Thirteen genotypes thrived on M2+ G, whereas two genotypes performed better on M2+ woS (Table 7).

**Table 7:** Surviving beech genotypes obtained in Exp I-III after being cultured up to 2 years

Experiment	Age	Provenance	N genotypes	Medium	Sugar source	Light intensity [µmol m^−2^ s^−1^]
I	14 years	19 (PL)	1	M2+ G	glucose	30
II	3 weeks	Grosshansdorf (DE)	10	M2+ woS	sugar-free	70
III	3 weeks	81009 (DE)	13	M2+ G	glucose	30
			2	M2+ woS	sugar-free	70

Cultures were maintained under standard PGC conditions with an subcultivation period of 6 weeks on M2+ medium. M2+ medium contained either no sugar source (sugar-free—M2+ woS) or 2% glucose (M2+ G)

## Discussion

### Experiment I

Overall, sample material from 22 out of the 23 provenances harvested could be successfully prepared for in vitro establishment. Both the date of sampling and the environmental conditions during bud break influenced the success of bud break on branches and therefore the ability to use actively growing shoots for in vitro establishment. In general, bud break initiation in branches under GH conditions (Fig. 1d), as well as sampling later in the season (May compared with February), increased the number of genotypes from which material could be collected for in vitro culture establishment. The low proportion of explants derived from February samples held under PGC conditions (11%) underscores the beneficial effect of bud break under GH conditions.

Since the use of actively growing shoots collected from the field is not recommended because of the high contamination rate [29, 54], we used actively growing shoots for in vitro establishment by sprouting under controlled conditions according to Barker et al. [30]. The containment strategy effectively reduced the loss of explants to contamination during in vitro establishment. Nevertheless, 52% of the prepared shoot tips and nodal segments developed contamination. As described in the literature, the risk of contamination increases with prolonged development of buds in situ on trees, but the in vitro shoot formation rate also increases (Table 3) [29, 39]. When all initial explants were considered, similar shoot formation rates were achieved for both the May and February samples (May: 5%; February: 4%). Therefore, the increased shoot formation in the May samples was offset by greater contamination loss.

The designated mixed samples were derived from provenances with successful bud break on branches sampled in February, and other trees from the same provenance were sampled in May. In this batch consisting of half-February and half-May samples, both an intermediate contamination rate and shoot formation rate were expected. Notably, while providing the expected intermediate contamination rate, the shoot formation rate was comparable to that of the May samples (18%, Table 3). This observation suggests a lack of a distinct correlation between the sampling date and shoot formation or indicates bias due to the experimental setup. Here, the small sample size per provenance in the May samples (N = 7) compared with the mixed samples (N = 28) is noteworthy. Although small sample sizes do not directly affect the shoot formation rate, they increase the risk of complete provenance loss, thereby influencing the overall shoot formation rate. Barker et al. highlighted material availability as a limiting factor for the in vitro culture establishment *of F. grandifolia* [30]. Additionally, in our approach, the availability of starting material was limited since the provenance trial trees were used for other physiological experiments and should not be excessively affected by sampling. Nevertheless, we aimed to include as many provenances as possible in this initial research approach. In our study, out of eight provenances with a sample size < 10 after contamination was removed, only provenances 16 (IT) and 21 (RO), both consisting of May samples, produced one shoot in vitro. Provenance 18 (IT), with the highest sample size and part of the mixed samples, also had the highest number of shoots after 12 weeks (14 shoots). This finding indicates a correlation between the success in establishing cultures and the bud break behaviour of branches after sampling, as the latter influences the availability of material that can be prepared. Therefore, increasing the sample size of provenances sampled in May by selecting genotypes with strong bud breaks on branches after sampling could further enhance the shoot formation rate of May samples. In addition, adapting the sterilization protocol, which may include antibiotics successfully used in E III, could avoid the high loss of explants to contaminations.

Our beech in vitro culture was established by sampling provenance trial trees to increase genetic diversity and assess provenance-specific responses in vitro [55]. This study pursued a novel and broad approach by aiming to establish 65 genotypes of 23 provenances in vitro, exceeding earlier studies that covered only three provenances [29, 30]. After 12 weeks, shoot formation was successfully induced in 13 of the 23 provenances (57%) sampled under in vitro conditions. A significant provenance effect on shoot formation was observed within the 13 provenances with a sample size ≥ 10, with provenance 5 (DE) showing the highest shoot formation rate of 80%. Additionally, provenances 18 (IT), 7 (DE), and 20 (PT) contributed particularly vigorously to the significant provenance effects on the probability of shoot formation according to logistic regression, as did provenances 13 (FR) and 19 (PL), which are rather poorly growing cultures. Although there are no extensive analyses of the diverse provenances of beech trees, Vieitez et al. [28] previously reported greater adventitious bud regeneration in German beech cultures than in Spanish cultures. Similarly, provenance-specific differences were found in the shoot formation of Douglas fir (*Pseudotsuga menziesii*) and Kashmir trees (*G. arborea*) or in in vitro regrowth after cryo-conservation in sessile oak (*Quercus petraea*) [48, 56, 57]. Since both the provenance and the sampling date significantly influence in vitro shoot formation, this factor must be considered in further statistical analyses. A third influencing factor known from the literature is the age of the tree used for culture establishment [29, 58]. In our approach, the age groups of the trees significantly overlap the provenances. Thus, tree age cannot be analysed independently of provenance.

Overall, various factors, such as provenance, genotype, age, trial location, sampling time, sample size, and bud break conditions, complicate statistical analyses of in vitro shoot formation. To further analyse the provenance effect and improve statistical analysis, a larger sample size per provenance is recommended for samples obtained under consistent conditions. This approach also addresses the potentially variable in vitro growth potential of various genotypes within a provenance [58, 59]. The sampling of branches in May with slightly opened buds with actively growing shoots developed under GH conditions is recommended when aiming to test as many provenances as possible for sustainability to establish them in vitro. When a provenance of interest exhibits recalcitrance to in vitro conditions, sampling in February and a second sampling of genotypes that succeed in the development of actively growing shoots in May are promising for maximizing the probability of successfully establishing in vitro cultures.

### Experiments II and III

For E II and E III, we utilized WS and ST of beech saplings for in vitro establishment, combining the advantages of young plant material with the ability to screen a high number of genotypes simultaneously. However, working with seedlings (one sample per genotype) poses significant challenges because of their high susceptibility to contamination loss, as observed in our study. As described for beech and European ash (*Fraxinus excelsior*), the type of explant strongly influences the contamination rate during in vitro establishment, with smaller explants and less exposure to the environment resulting in lower contamination rates [38, 60]. The higher contamination rates in WS than in ST can be attributed to the larger surface area, greater degree of soil contact, and greater abundance of endophytic bacteria. The loss of explants due to fungal contamination was minimized by using ST instead of WS, without significantly affecting shoot formation during the first 12 weeks of in vitro culture (Tables 4 and 5). Bacterial contamination was a serious issue in the seedling-derived explants, affecting 32% (E II) to 41% (E III) of the ST explants. The use of antibiotics effectively eliminated bacterial contamination during in vitro establishment without significantly impacting the shoot formation rate, shoot length, or shoot number (Tables 5 and 6). The low phytotoxicity of streptomycin, gentamicin, and cefotaxime shown in date palm or poplar in in vitro culture was confirmed for *F. sylvatica* [61–63].

Interestingly, shoot formation occurred on M0 without phytohormones in both experiments, contrary to the literature [30, 45]. The shoot formation rate on M0 was greater than that on hormone-containing media (M2.2 and M2.3) during the first 8 weeks (Fig. 3i). This, alongside differences in leaf morphology (Fig. 3d–f), may be explained by the influence of phytohormones on leaf primordium development [64, 65]. As previously described for *Fagus* spp. [29, 30], the viability of in vitro formed shoots began to decline within the first weeks of culture in E II (Fig. 3h). Consequently, differences in shoot formation rates between the media converged after 12 weeks. Similar to findings in *F. grandifolia* ST explants [30], while the shoot formation rate, number, and length were not significantly affected, M2 medium supplemented with 0.89 µM BAP and 0.27 µM NAA led to more vigorous growth and darker green leaves. However, chlorosis, hyperhydricity, and callus formation—signs of excessive cytokinin use—were increased in shoots initially cultured on high phytohormone-containing media M2.2 and M3 [30].

To address vitality loss and growth issues indicated by chlorosis (Fig. 3f) and resting bud formation (Fig. 3h), which culminated in explant browning within the first 12 weeks of culture in E II (Fig. 3i), we supplemented M2 culture media with additional FeNaEDTA and MgSO_4_ (M2+) in E III. Increases in iron or magnesium in culture media have been shown to positively influence microcutting growth in other species [66–71]. In our study, no significant differences in shoot formation rates were found between the individual culture media. However, shoot formation was significantly higher on M2 + in E III than on WPM-based media in E II (73% vs. 51%; Fig. 4c). The requirement for M2+ medium for micropropagation after 12 weeks and the higher proportion of genotypes that could be further cultivated after week 12 in E III (27%) than in E II (8%; Table 7) support a more suitable micronutrient composition in both FeNaEDTA- and MgSO_4_-supplemented media for beech in vitro growth after the first subculture intervall.

The seed supplier had a significant influence on shoot formation rates in E III (Fig. 4b). Low germination rates, likely tied to poor seed viability and seasonal factors in beech [72], negatively impact in vitro shoot formation. Thus, seasonal fluctuations in germination rate should be considered, with March–May and September–November showing promising results.

In general, the addition of phytohormones to culture media has a fundamental effect on the growth and differentiation of in vitro*-grown* plants, as well as on their physiological processes [73]. In our approaches, phytohormones were required for multiple shoot induction (Fig. 3j), highlighting their role beyond the initial culture phase. While bud break at cotyledonary nodes occurred in WS explants cultured on hormone-containing media in E II (Fig. 3b), phytohormone supplementation did not result in significant differences in shoot numbers between WS and ST, likely because apical dominance limits lateral shoot development [74]. Shoot multiplication occured through axillary branching (Fig. 3g), resulting in a median of three shoots per explant, regardless of the hormone concentration (Fig. 3j). The use of hormone-containing media for ST cultivation over the entire period of 12 weeks in E II led to significantly higher number of shoots derived from a single explant, compared to ST explants cultivated on hormone-free medium M0 for the first six weeks in E III (Fig. 4e). This may be due to the shorter time on hormone-containing media necessary for multiple shoot induction (Fig. 3j), as supported by the greater variability in the number of shoots on M2+ than on M0 (Fig. 4e).

The length of the shoots formed was unaffected by phytohormone concentrations in E II (Fig. 3k). However, the shoot length in E II (median length of 10 mm) was significantly greater than that in E III (5 mm, Fig. 4d) after 12 weeks. This difference may highlight the subculture interval of 4 weeks in E II versus 6 weeks in E III as another relevant factor. Vieitez et al. reported a negative effect of less frequent medium changes on shoot elongation in *F. sylvatica* [28]. For *F. grandifolia*, Barker et al. reported 18 mm shoot length at 12 weeks, with medium changes every three weeks [30]. Reducing the subculture interval could therefore be advisable, although this should be verified in future experiments.

Another factor influencing the shoot formation rate, multiplication, and length may be the provenance of the seeds used in E II and E III. Provenance-specific influences on micropropagation abilities are indicated by the results obtained in E I and have been previously described for adventitious bud regeneration in beech [28]. Genotype-specific influences on shoot multiplication have also been documented [47]. Thus, provenance-specific differences between E II and E III should be considered when validating the effects of culture medium composition. Consequently, seed provenance, quality, and seedling vitality (timing of in vitro culture establishment) influence shoot formation rates, multiplication, and length within the first 12 weeks. Even so, the use of seedlings resulted in relatively high shoot formation rates in two independent experiments (51% and 73%) compared to those reported by Vieitez et al., Meier and Reuther, and Hazubska-Przybyl et al. [28, 29, 38]. At least one genotype from each provenance was successfully propagated in vitro (Table 7).

### Micropropagation—Effects of the carbon source and light intensity

In this study, when seedlings (E III) were used for culture establishment, no significant difference in growth was observed between the M2+ medium with and without sucrose (Table 6). Switching from M2+ to M2+ woS for cultures showing chlorosis or growth arrest (evidenced by resting bud formation) improved vitality and promoted bud sprouting (Fig. 5i). Accordingly, for some woody plant species, autotrophic growth on sugar-free media in combination with increased light intensity can be equal to or even surpass mixotrophic growth [52]. Sugar-free media should force plantlets to rely on their photosynthetic apparatus [75].

Beech trees are highly shade tolerant and grow best under low light conditions as young plants. Since chlorosis and resting buds occurred under 70 µmol m⁻^2^ s⁻^1^ on M2+ medium, this light intensity may have been sufficient to reduce the vigor of the cultures [76]. A lower light intensity during subsequent cultivation on M2+ G medium resulted in darker green leaves. Additionally, the cultures were maintained at 70 µmol m⁻^2^ s⁻^1^ on the sugar-free medium M2+ woS, where they grew well, as sugar-free media can increase high light tolerance in plant cultures [77].

Excessive bacterial growth in woody plant in vitro cultures is a well-known issue affecting plant vitality [78]. As previously reported for other tree species [52], without a carbon source in the medium M2 + woS, we achieved reduced bacterial overgrowth, allowing an increased number of beech genotypes to be cultured (Fig. 5f, g, j, Table 7). Although the use of sugar-free media in our experiments successfully addressed the negative observations, further optimization was necessary to improve shoot elongation.

Different saccharides have physiological and morphogenetic influences on in vitro cultures of trees [73, 79]. Cuenca and Vieitez [40] reported that, depending on the genotype, shoot elongation in *F. sylvatica* and *F. orientalis* can be enhanced with the addition of glucose to the culture medium, which is comparable to observations with in vitro cultures of *Paulownia tomentosa* [79]. Thus, glucose was tested as a carbon source in the medium (M2+ G) to address the poor shoot elongation on M2 + and M2 + woS. Genotypes without bacterial issues exhibited the most vigorous growth on M2+ G. Therefore, M2+ G was used in prolonged in vitro culture (Table 7). Consistent with the literature, increased callus formation at the shoot base was observed with glucose (Fig. 5k, l), possibly because glucose can act as a signalling molecule, promoting cell division and differentiation [40, 80]. Additionally, adventitious bud formation was noted, with newly emerging buds showing no delay in bud break, in contrast to M2+ plants (Fig. 5d, k). Since hexoses such as glucose make up a large portion of the sugars in beech leaves and shoots, their bioavailability appears to be relatively high, which could lead to improved plant growth on M2+ G [81]. Although the microbiome of European beech has not yet been studied for preferred in vitro parameters, it is known from other plant species that endophytic bacteria have specific carbohydrate preferences [82]. A possible adaption of endophytic bacteria to glucose could contribute to the intense bacterial growth observed for some genotypes on the glucose-containing M2+ G medium.

In this study, shoot formation was initially achieved in 57% of 23 provenances in vitro, but after two years, only one genotype from provenance 19 (PL; E I) remained (4% of the initial 23 provenances). In seedlings, the proportion of genotypes growing in vitro decreased from 51 to 8% (E II) or from 73 to 27% (E III). Maintaining long-term beech cultures has proven challenging in previous studies [28–30]. Despite optimizing nutrient composition, carbon sources, and light intensity, further adjustments are needed for long-term culture success, e.g., phytohormone supplementation or shorter subcultivation intervals. To draw conclusions about the effect of light intensity and the carbon source on the growth of in vitro shoots, further experiments would need to be conducted.

### Rooting and *ex vitro* acclimatization of microcuttings

Rooting and greenhouse acclimatization are essential for the use of in vitro cultivated microcuttings for controlled experiments. These last steps are known to be critical: rooting was successful in 57–90% of the cultures in earlier studies [28, 36, 37]. The survival rate of in vitro rooted plantlets reached 72% after the transfer to soil in humidity tunnels [28]. However, unsuccessful acclimatization has also been reported [30]. Both root induction by IBA dip and transfer to soil and acclimatization were successful in our initial proof-of-principle trial (Fig. 5g, j). Larger trials must be conducted for further validation and optimization. Notably, not all explants are equally suitable. In particular, shoots that develop from callus-like tissue may root poorly because of weak vascular connections [83, 84].

## Conclusions

The long lifespan of forest trees and their relatively slow growth can turn research into a multi-generational project. New approaches to establish in vitro cultures of forest trees, such as beech, benefit from previous in vitro work and can provide new contributions. This opens new research avenues and can support the adaptation of beech to climate change.

Our protocol for establishing in vitro beech cultures offers the possibility of utilizing beech provenance trials. If sprouting occurs under controlled conditions, actively growing shoots can be used to initiate a culture. Based on our results, shoot formation can be expected in 13.3% of explants, representing 57% of provenances. If possible, the loss of explants due to contamination should be addressed by increasing sample sizes per provenance when sampling branches in May. The use of antibiotics is recommended to reduce bacterial contamination. If certain provenances prove challenging for in vitro culture, the described provenance-dependent growth potential should be considered.

The high shoot formation rate of up to 94% makes it advisable to use seedlings as starting material for establishing vigorous cultures, especially for future methodological tests or large-scale screenings. From this starting material, the use of only ST is sufficient. However, the quality of beechnut is crucial in this process.

In long-term cultures, reducing the outgrowth of endophytic bacteria or revitalization after growth arrestment may be achieved by using sugar-free culture media. Additionally, supplementation with FeNaEDTA and MgSO_4_ is suitable for maintaining healthy explants. Future research should continue to optimize the cultivation process, as growth rates are still not satisfactory. Furthermore, the individual factors presented here for the establishment and cultivation of in vitro beech serve as suitable starting points for further focused research.

## Supplementary Information

Additional file 1. Provenances of the beech genotypes used for *in vitro* culture establishment (Experiment I) and their climatic conditions. The average annual temperature and annual precipitation are based on climate data from Worldclim 2 for the period of 1970–2000. Provenances are numbered according to Table 2. Country abbreviations according to ISO 3166 1 alpha2. NP – National park.

Additional file 2. Effect of tree age on *in vitro* shoot formation within a 12-week culture period. Within the heatmap, all provenances of the same age are grouped into specific age categories (labelled “age of samples trees [years]”). All age groups highlighted in blue are significantly different from each other, as determined by pairwise Fisher’s exact test with a significance level of p ≤ 0.05.

Additional file 3. Evaluation of beech seedlings 12 weeks after *in vitro* culture establishment. Five different media (M0, M2, M2.1, M2.2, M3) were tested using whole shoots (WS) or shoot tips (ST) of seedlings. The WS and ST or media were either separately evaluated or combined (pooled). The percentages of explants exhibiting shoot formation, bacterial or fungal contamination, resting bud formation, and chlorosis are given. The median was calculated for shoot length and the number of shoots derived from one explant. Values labelled with different letters indicate significant differences between media/explant types according to pairwise Dunn’s test for the number of shoots or shoot length and Pearson’s chi‒squared test for bacterial contamination, fungal contamination, resting bud formation, and chlorosis with p ≤ 0.05. IQR – interquartile range.

## Data Availability

All data generated or analysed during this study are included in this published article or its supplementary information files. The raw data are available from the corresponding author upon reasonable request.
