# Supplementary figures and images for: Benefiting from the past: establishing in vitro culture of European beech (Fagus sylvatica L.) from provenance trial trees and seedlings
Source: Plant Methods. 2025 Mar 7;21:31. doi: 10.1186/s13007-025-01350-3 (PMC11887157; doi:10.1186/s13007-025-01350-3)

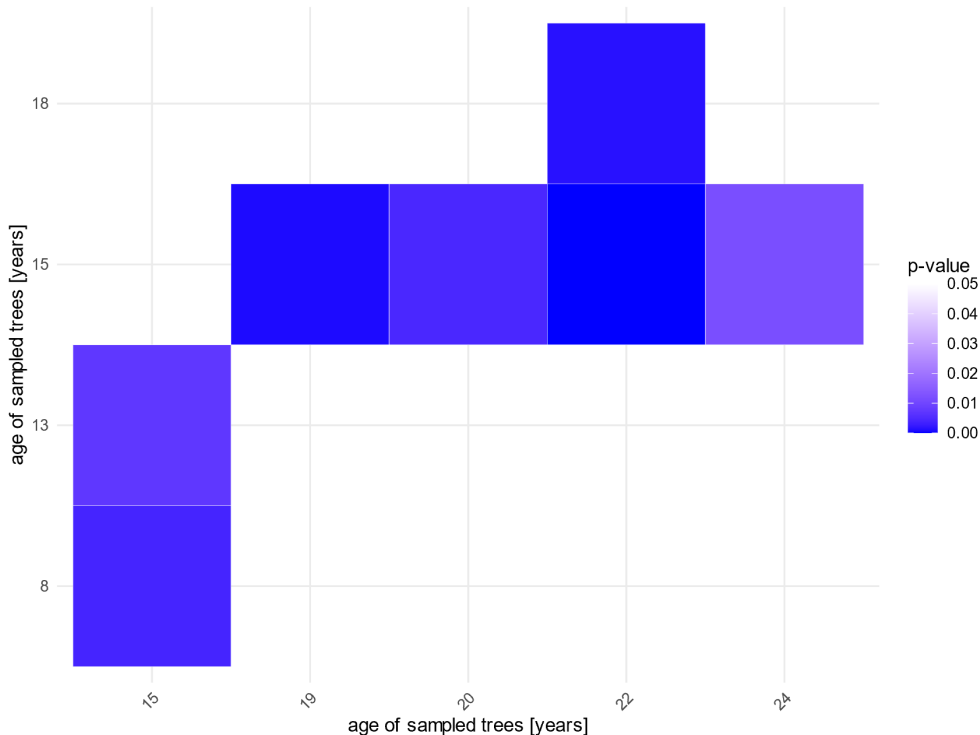

Supplement: Supplementary file 2 — Additional file 2. Effect of tree age on in vitro shoot formation within a 12-week culture period. Within the heatmap, all provenances of the same age are grouped into specific age categories (labelled “age of samples trees [years]”). All age groups highlighted in blue are significantly different from each other, as determined by pairwise Fisher’s exact test with a significance level of p ≤ 0.05. [file 13007_2025_1350_MOESM2_ESM.pdf]
